# Distribution of Immunomodulation, Protection and Regeneration Factors in Cleft-Affected Bone and Cartilage

**DOI:** 10.3390/diagnostics14192217

**Published:** 2024-10-04

**Authors:** Mārtiņš Vaivads, Māra Pilmane

**Affiliations:** Department of Morphology, Institute of Anatomy and Anthropology, Rīga Stradiņš University, Kronvalda Boulevard 9, LV-1010 Riga, Latvia; mara.pilmane@rsu.lv

**Keywords:** cleft lip and palate, bone, hyaline cartilage, Gal-10, NF-κB p65, HSP60, HSP70, LL-37, Col-I, BMP-2/4

## Abstract

Background: Craniofacial clefts can form a significant defect within bone and cartilage, which can negatively affect tissue homeostasis and the remodeling process. Multiple proteins can affect supportive tissue growth, while also regulating local immune response and tissue protection. Some of these factors, like galectin-10 (Gal-10), nuclear factor kappa-light-chain-enhancer of activated B cells protein 65 (NF-κB p65), heat shock protein 60 (HSP60) and 70 (HSP70) and cathelicidin (LL-37), have not been well studied in cleft-affected supportive tissue, while more known tissue regeneration regulators like type I collagen (Col-I) and bone morphogenetic proteins 2 and 4 (BMP-2/4) have not been assessed jointly with immunomodulation and protective proteins. Information about the presence and interaction of these proteins in cleft-affected supportive tissue could be helpful in developing biomaterials and improving cleft treatment. Methods: Two control groups and two cleft patient groups for bone tissue and cartilage, respectively, were organized with five patients in each group. Immunohistochemistry with the semiquantitative counting method was implemented to determine Gal-10-, NF-κB p65-, HSP60-, HSP70-, LL-37-, Col-I- and BMP-2/4-positive cells within the tissue. Results: Factor-positive cells were identified in each study group. Multiple statistically significant correlations were identified. Conclusions: A significant increase in HSP70-positive chondrocytes in cleft patients could indicate that HSP70 might be reacting to stressors caused by the local tissue defect. A significant increase in Col-I-positive osteocytes in cleft patients might indicate increased bone remodeling and osteocyte activity due to the presence of a cleft. Correlations between factors indicate notable differences in molecular interactions within each group.

## 1. Introduction

Cleft lip and palate are relatively frequent congenital anomalies that have an incidence of 1 per 600 to 800 newborns in the general population [1]. Orofacial clefts in humans are a spectrum of disorders that develop due to improper fusion of facial folds within the 4th–12th week of embryonic development and can affect not only the soft mucosal tissue, but also the underlying bone and cartilage [2]. Cleft formation negatively affects the growth and development of maxillary bone and cartilage causing defects in facial morphology in affected children [3,4]. Supportive tissue growth can be regulated by multiple factors that affect regeneration and regulation of local immune response within this tissue.

Galectin-10 (Gal-10) is a protein belonging to the galectin protein family due to its structural similarity to other proteins in this group [5]. Gal-10 can be mainly found in human eosinophilic leukocytes [6] and has been associated with pathologies where eosinophilic leukocyte involvement has been notified like allergies, asthma, helminth parasite infections and others [5]. Gal-10 is needed for the differentiation and growth of eosinophils [7]. Galectins can also be found in cartilage and bone in the case of osteoarthritis where tissue dysregulation and excessive inflammation are present [8]. Gal-10 involvement in orofacial supportive tissue growth has not been well documented.

Nuclear factor kappa-light-chain-enhancer of activated B cells protein 65 (NF-κB p65) has been described as a factor involved in inflammation and developmental processes. An increase in NF-κB p65 has been associated with chronic inflammatory and degenerative disorders [9]. NF-κB p65 in interaction with other proteins inhibits apoptosis [10]. NF-κB p65 activation by proinflammatory cytokines affects supportive tissue growth—activation of NF-κB p65 represses osteoblast differentiation and bone growth [11,12]—while in cartilage, it represses chondrocyte apoptosis [13]. The presence of NF-κB p65 in cleft-affected bone and cartilage has not been well described in previous research.

Heat shock protein 60 (HSP60) is a chaperonin that typically can be found in mitochondria, but it has been identified in the cell cytoplasm, plasma membrane or even extracellular space [14]. HSP60 regulates protein folding and protein homeostasis in the mitochondria together with other chaperonins [15], but other functions have also been attributed. It has been previously described that HSP60- and HSP60-derived peptides have anti-inflammatory and immunomodulating action [16,17]. HSP60 in bone tissue has protective properties against stressors [18,19], while in cartilage disorders like osteoarthritis, HSP60 decreases the loss of the cartilage matrix [20]. HSP60’s involvement in cleft-affected supportive tissue has not been well documented in previous research.

Heat shock protein 70 (HSP70) is a chaperonin that regulates protein folding, aggregation and general protein homeostasis in cells and, together with other heat shock proteins, forms a complex network of interactions [21,22]. HSP70 protects other proteins from stress-induced denaturation [23,24], but other functions have also been described. In bone tissue, HSP70 protein 8 non-covalently binds with monoclonal nonspecific suppressor factor-β (MNSFβ), which can promote receptor activator of nuclear factor-kappa beta (RANKL) induction of osteoclastogenesis and subsequent increase in bone resorption [25]. HSP70 is involved during endochondral ossification and inhibits vascular endothelial growth factor (VEGF) synthesis, which regulates the growth of blood vessels during the ossification process [26]. In cartilage, HSP70 levels increase in the presence of stressors [27]. An increase in anti-HSP70 antibodies in women who later had given birth to children with cleft lip and palate has been described but the exact mechanism of possible interaction during cleft formation remains unclear [28]. Evaluation of HSP70 in cleft-affected supportive tissue has not been well described in previous research.

Cathelicidin (leucine leucine-37 or LL-37) is a 37 amino acid cationic peptide that is an important factor for innate immune response [29]. LL-37 has immunomodulating action—it can promote inflammation in some tissues, while in others, it has anti-inflammatory action [30]. LL-37 promotes osteogenic differentiation of mesenchymal stem cells while also stimulating bone regeneration and local antibacterial protection [31,32,33]. Regenerating and antibacterial effects of LL-37 have been studied in articular cartilage [34,35]. LL-37 has not been well studied in cleft-affected supportive tissue.

Type I collagen (Col-I) is the most common type of collagen that is typically found in many types of connective tissue [36]. It is an important structural protein in bone tissue and is essential for correct bone growth and regeneration [37]. Col-I is typically not found in most hyaline cartilage [38], but studies have shown that some Col-I can be detected in hyaline cartilage [39,40], which affects the biomechanical properties of this tissue. Col-I has additional functions in regulating bone cell growth and differentiation [41]. It functions as a ligand for receptors that stimulate osteoblast differentiation and inhibit osteoclast activity [42]. The expression of genes that are needed for Col-I synthesis is downregulated in osteocytes during their differentiation from osteoblasts [43], but osteocytes can also produce Col-I to maintain bone homeostasis [44]. Col-I production in chondrocytes has also been demonstrated in animal models [45]. Increased Col-I protein production has been detected in the connective tissue of cleft lip and palate patients, indicating potential issues in wound healing and scarring after cleft-correcting surgery [46], while the presence of Col-I in cleft-affected bone tissue and cartilage cells has not been well documented in previous studies.

Bone morphogenetic proteins 2 and 4 (BMP-2/4) are highly involved in supportive tissue homeostasis regulation. BMP-2 is an important growth factor during bone tissue formation and osteoblast activation [47]. BMP-4 is a necessary growth factor during skeletogenesis and during different organ system development [48], and it promotes osteogenetic differentiation of mesenchymal stem cells [49]. The BMP signaling pathway regulates bone remodeling [50] and enhances cartilage growth [51]. Both BMP-2 [52] and BMP-4 [53] stimulate chondrogenesis. BMP-2/4 have been studied in cleft-affected supportive tissue [54], but their exact interactions with other previously mentioned immunomodulating and protective factors in cleft-affected supportive tissue have not been well studied.

The main aim of this study is to describe and compare the presence and relative distribution of Gal-10, NF-κB p65, HSP60, HSP70, LL-37, Col-I and BMP-2/4 protein-containing bone and cartilage cells by using immunohistochemistry in both relatively healthy control supportive tissue and cleft-affected bone and cartilage. Understanding the distribution and presence of these factors, as well as the correlations between them in cleft-affected bone and cartilage, could provide additional information about differences between relatively normal and cleft-affected supportive tissue, differences or similarities in healing, regeneration and immune response after surgical intervention. This information could provide a clearer perception of new directions of treatment and biomaterial development regarding cleft-affected supportive tissue repair.

## 2. Materials and Methods

### 2.1. Characterization of Patients and Controls

Tissue samples that were gathered and used for patient groups and control groups were gathered in the Cleft Lip and Palate Centre of the Institute of Stomatology of Riga Stradins University (RSU). Each tissue sample was donated for research on voluntary agreement from the parents of patients. Tissue samples were evaluated in the RSU Institute of Anatomy and Anthropology. RSU’s Research Ethics Committee issued the necessary permissions (first permission dated 22 May 2003 and second permission Nr. 5 dated 28 June 2018) to allow us to perform this study. This research work was conducted based on the Declaration of Helsinki regulations of 1964.

Supportive tissue recovered from cleft patients was subdivided based on the available tissue type taken during the third stage of cleft-correcting surgery (alveolar process osteoplasty and nasal septum correction; each cleft patient had undergone primary lip plastic surgery and secondary soft palate plastic surgery previously at an earlier age) [55] into cleft-affected bone tissue group (5 patients) and cleft-affected cartilage tissue group (5 patients). The inclusion criteria for each patient group were the following: diagnosis of non-syndromic cleft lip and palate, no other orofacial pathology detected, cleft-correcting surgery has been performed during mixed dentition age. Only excess tissue (approximately 1 mm^3^) not needed for cleft surgical repair was taken and used for research to prevent harm to the patients. The average age of patients in the cleft-affected bone tissue was 10 years and 9 months (ranging from 6 years 7 months to 14 years 5 months), while patients in the cleft-affected cartilage group, on average, were 9 years and 7 months old (ranging from 6 years and 7 months to 11 years and 6 months). Four out of five patients in the cleft-affected bone tissue group were male, while one patient was female. Similarly, four out of five patients in the cleft-affected cartilage group were male and one patient was female.

Bone tissue and cartilage tissue from controls were gathered from individuals of mixed dentition age, while both primary and permanent teeth are present typically between the ages of 6 to 12 years [56]. Inclusion criteria for control group tissue were the following: no orofacial clefts in anamnesis or family history and no other pathological processes present in supportive tissue (either bone or cartilage) like inflammation, tissue degeneration or malignancy. The tissue material for bone tissue controls (5 patients—3 male and 2 female; all mixed dentition age) was gathered from alveolar bone during tooth extraction surgery not related to cleft lip and palate, which was taken during routine surgery in RSU Institute of Stomatology. The tissue used for cartilage controls (5 patients—4 male and 1 female; all mixed dentition age) was gathered from the tracheal cartilage of cadavers in the RSU Institute of Anatomy and Anthropology.

### 2.2. Tissue Preparation, Routine Staining and Immunohistochemistry

The tissue material of patients and controls was fixated directly after retrieval in Stefanini (Zamboni) solution (12773.02500, ZAMBONI solution-2.500 ml, MORPHISTO Ltd., Offenbach am Main, Germany) and further prepared in the RSU Institute of Anatomy and Anthropology Laboratory of Morphology. Later, Tyrode’s solution was used for the washing procedure for 24 h. The tissue material was dehydrated in an alcohol solution and, afterward, degreased with xylene for 30 min. Next, the specimens were embedded in paraffin and the fully hardened blocks were cut into several 5 µm thick sections using a semi-automatic rotary microtome (Leica RM2245, Leica Biosystems Richmond Inc., Richmond, IL, USA). After sections were fixed on slides, deparaffinization was performed. First, tissue was routinely stained with hematoxylin (05-M06002, Mayer’s Hematoxylin, Bio Optica Milano S.p.A., Milan, Italy) and eosin (05-B10003, Eosin Y alcoholic solution, Bio Optica Milano S.p.A., Milan, Italy) to provide a general overview. Second, immunohistochemistry was performed to determine the presence of Gal-10, NF-κB p65, HSP60, HSP70, LL-37, Col-I and BMP-2/4 protein-containing cells in both patient and control groups.

Samples of supportive tissue (bone and cartilage) were prepared for immunohistochemical investigation and light microscopy by employing standard streptavidin and biotin immunostaining methods [57] to identify immunopositive cells within bone and cartilage tissue of patients and controls. Immunohistochemistry with different antibodies was performed in accordance with the technical specifications given by antibody manufacturers. The following antibodies were used for the identification of factor-positive bone and cartilage cells: Gal-10 antibodies (orb157023, rabbit, polyclonal, dilution 1:200, Biorbyt Ltd., Cambridge, UK); NF-κB p65 antibodies (orb37069, rabbit, polyclonal, dilution 1:100, Biorbyt Ltd., Cambridge, UK); HSP60 antibodies (sc-1052, goat, polyclonal, dilution 1:100, Santa Cruz Biotechnology, Inc., Dallas, TX, USA); HSP70 antibodies (33-3800, mouse, monoclonal (MB-H1), dilution 1:100, Invitrogen, Waltham, MA, USA); LL-37 antibodies (sc-166770, mouse, monoclonal (D-5), dilution 1:100, Santa Cruz Biotechnology, Inc., Dallas, TX, USA); Col-I antibodies (NB600-408, rabbit, polyclonal, dilution 1:100, Novus Biologicals, LLC, Centennial, CO, USA); and BMP-2/4 antibodies (AF355, goat, polyclonal, dilution 1:100, R&D Systems, Inc., Minneapolis, MN, USA).

The nonparametric evaluation of the relative frequency of factor-positive cells within bone and cartilage was performed with the semiquantitative counting method [58]. The supportive tissue (bone or cartilage) in each slide was evaluated under a light microscope in 5 separate visual fields for assessment. Slide evaluation was performed by two independent scientists. The following semiquantitative denominations were used: 0—no immunoreactive cells in the visual field (0.0%); 0/+—a rare occurrence of immunoreactive cells in the visual field (0.0–12.5%); +—a few immunoreactive cells in the visual field (12.5–25.0%); +/++—a few to moderate number of immunoreactive cells in the visual field (25.0–37.5%); ++—a moderate number of immunoreactive cells in the visual field (37.5–50.0%); ++/+++—a moderate to numerous number of immunoreactive cells in the visual field (50.0–62.5%); +++—numerous immunoreactive cells in the visual field (62.5–75.0%); +++/++++—numerous to abundant number of immunoreactive cells in the visual field (75.0–87.5%); and ++++—an abundance of immunoreactive cells in the visual field (87.5–100.0%).

Slide microphotographs were obtained with a Leica DC 300F digital camera (Leica Microsystems Digital Imaging, Cambridge, UK), which later were processed with the Image Pro Plus program (Media Cybernetics, Inc., Rockville, MD, USA).

### 2.3. Statistical Methods

Descriptive and analytical statistics methods were employed for data analysis. The semiquantitative count of factor-containing cells within each visual field was evaluated with descriptive statistics methods, including median value calculation. Analytical statistics methods like the Mann–Whitney U test and Spearman’s correlation coefficient calculation were used. The Mann–Whitney U test was used to determine the statistical significance in the number of factor-positive cells between the control group and the cleft patient group for each supportive tissue type (bone tissue or cartilage). To determine correlations between the number of factor-containing cells, Spearman’s correlation coefficient (r_s_) was interpreted with the following values: r_s_= 0.0–0.2—very weak correlation, r_s_= 0.2–0.4—weak correlation, r_s_= 0.4–0.6—moderate correlation, r_s_= 0.6–0.8—strong correlation and r_s_= 0.8–1.0 very strong correlation. Statistical evaluation was performed with Statistical Product and Service Solutions (SPSS) Statistics version 29.0.0.0 (IBM Company, Chicago, IL, USA). A *p* value of <0.05 was employed to determine the statistical significance of every calculation, where needed.

## 3. Results

### 3.1. Routine Hematoxylin and Eosin Staining

**Figure 1:**
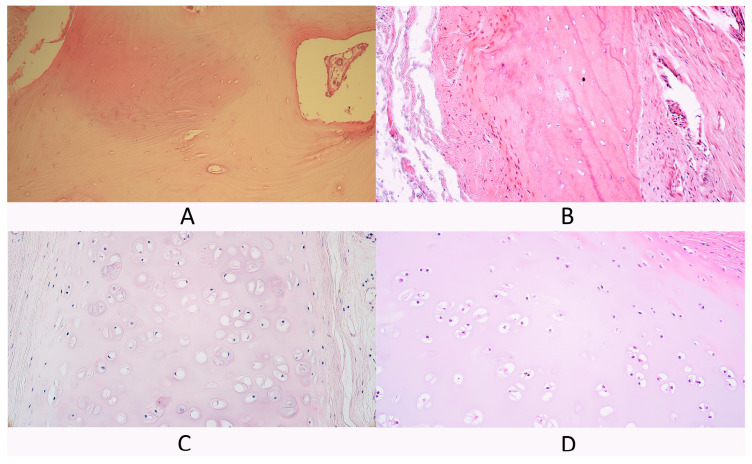
Hematoxylin and eosin (H&E) stained control tissue and cleft-affected supportive tissue. (**A**) Control bone tissue, H&E, 200×. (**B**) Cleft-affected bone tissue with surrounding periosteum, H&E, 200×. (**C**) Control cartilage tissue with surrounding perichondrium, H&E, 200×. (**D**) Cleft-affected cartilage tissue, H&E, 200×.

Bone tissue control group slides contained typical spongy bone trabeculae with some periosteum fragments (Figure 1A).

In slides from the cleft-affected bone tissue group bone fragments with some surrounding connective tissue were presented in all slides. Spongy bone trabeculae were visible in all slides. Cleft patient spongy bone was similar in structure to control tissue spongy bone (Figure 1B). Additionally, some compact bone fragments were detected in two slides. Compact bone fragments contained osteons, some of them had dilated Haversian canals, while, in one slide, chaotically oriented bone lamellae and dilated osteocyte lacunae were found. In some cleft-affected bone tissue slides, periosteum fragments were present with blood vessels and some nerve fiber bundles.

The cartilage tissue control group had hyaline cartilage plates with perichondrium (Figure 1C). Additionally, in two control patients, some mucoserous glands were also visible near the perichondrium.

Cleft-affected cartilage tissue slides had relatively normal hyaline cartilage tissue similar to the control group (Figure 1D). Most slides had some perichondrium fragments, while, in two slides, perichondrium was missing.

### 3.2. Immunohistochemistry

#### 3.2.1. Gal-10

**Figure 2:**
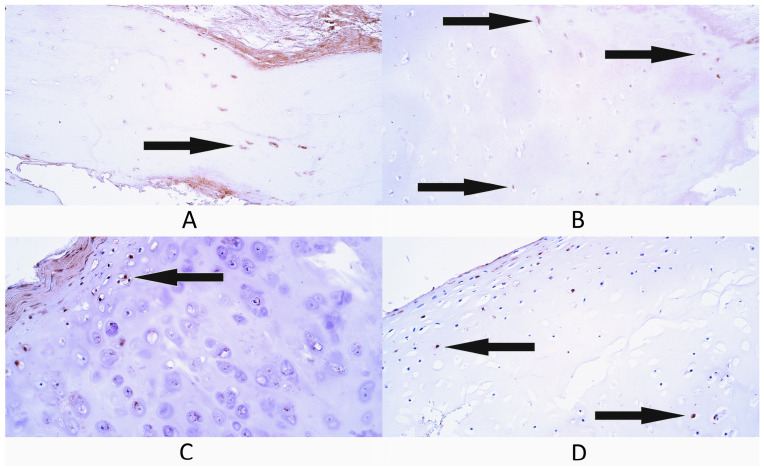
Immunohistochemistry (IMH) of galectin-10 (Gal-10)-containing cells in control and cleft-affected supportive tissue. (**A**) A few Gal-10-positive osteocytes (arrow) in control group bone tissue, Gal-10 IMH, 200×. (**B**) A few Gal-10-positive osteocytes (arrows) in cleft-affected bone tissue, Gal-10 IMH, 200×. (**C**) A few Gal-10-positive chondroblasts and chondrocytes (arrow) in the control group hyaline cartilage, Gal-10 IMH, 200×. (**D**) A rare occurrence of Gal-10-positive chondrocytes (arrows) in cleft-affected hyaline cartilage, Gal-10 IMH, 200×.

In the bone tissue control group, there were a few (+) Gal-10-containing osteocytes as the median value within this group (Figure 2A). Three patients had a few (+) Gal-10-containing osteocytes, while two patients had a rare occurrence (0/+) of galectin-containing osteocytes.

In cleft-affected bone tissue, the median number of Gal-10-containing osteocytes was also a few (+) (Figure 2B). One patient had a moderate number (++) of galectin-containing osteocytes, one patient had a few to moderate (+/++), while three patients had a few (+) Gal-10-containing osteocytes.

In cartilage tissue controls, there was a moderate (++) number of Gal-10-containing chondrocytes and chondroblasts as the median value (Figure 2C). One patient had moderate to numerous (++/+++) galectin-containing cartilage cells, three patients had a moderate (++) number, while one patient had a few to moderate (+/++) number of Gal-10-containing cartilage cells.

In cleft-affected cartilage tissue, the median number of Gal-10-containing cartilage cells was few to moderate (+/++) (Figure 2D). One patient had numerous (+++) Gal-10-positive cartilage cells, one patient had a moderate number (++), but three patients a few to moderate (+/++) numbers.

Statistically significant differences were not found in the number of Gal-10-positive cells between bone tissue controls and cleft-affected bone tissue (*p* = 0.095) and between cartilage tissue controls and cleft-affected cartilage (*p* = 0.548).

#### 3.2.2. NF-κB p65

**Figure 3:**
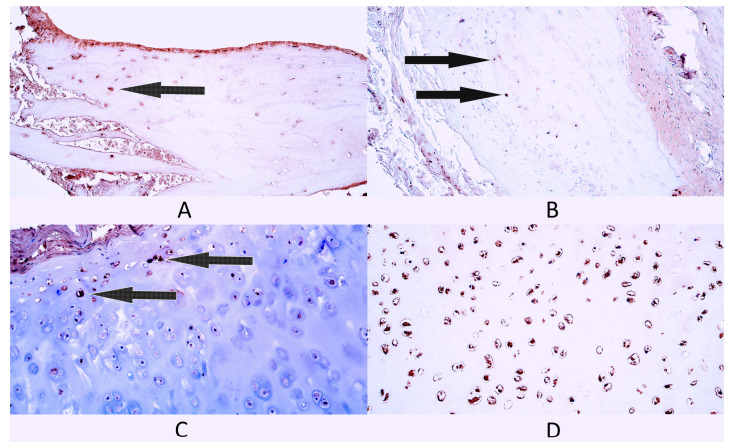
Immunohistochemistry of nuclear factor kappa-light-chain-enhancer of activated B cells protein 65 (NF-κB p65)-containing cells in control and cleft-affected supportive tissue. (**A**) A few NF-κB p65-positive osteocytes (arrow) in control bone tissue, NF-κB p65 IMH, 200×. (**B**) A few NF-κB p65-positive osteocytes (arrows) in cleft-affected bone tissue, NF-κB p65 IMH, 200×. (**C**) Few to moderate number of NF-κB p65-positive chondrocytes and chondroblasts (arrows) in control hyaline cartilage, NF-κB p65 IMH, 200×. (**D**) Numerous NF-κB p65-positive chondrocytes in cleft-affected cartilage, NF-κB p65 IMH, 200×.

Bone tissue controls had a rare occurrence (0/+) of NF-κB p65-positive bone cells as the median value within this group. Three control patients had a rare occurrence (0/+) of NF-κB p65-containing cells, while two patients had a few (+) factor-positive cells (Figure 3A).

The median number of NF-κB p65-containing cells in cleft-affected bone tissue was a few (+) (Figure 3B). Three patients had a few (+) NF-κB p65-positive cells, while two patients had a few to moderate (+/++) number of NF-κB p65-positive cells.

Cartilage tissue controls had a moderate (++) number of NF-κB p65-containing cells. Two patients had few to moderate (+/++) factor-positive cells (Figure 3C), two patients had a moderate (++) number and one patient had moderate to numerous (++/+++) NF-κB p65-positive cartilage cells.

The cleft-affected cartilage tissue group had numerous (+++) NF-κB p65-positive cartilage cells as the median value. Four patients had numerous (+++) NF-κB p65-positive cells (Figure 3D), while one patient had a few (+) factor-positive cells.

Statistically significant differences were not found in the number of NF-κB p65-positive cells between bone tissue controls and cleft-affected bone tissue (*p* = 0.056) and between cartilage controls and cleft-affected cartilage (*p* = 0.151).

#### 3.2.3. HSP60

**Figure 4:**
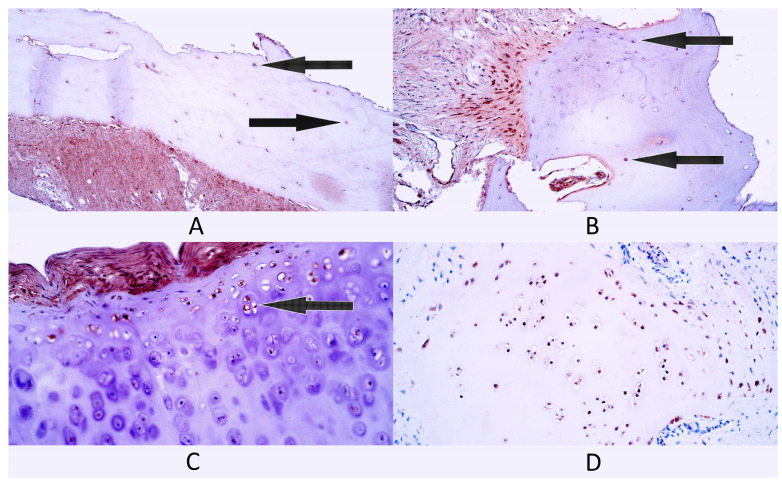
Immunohistochemistry of heat shock protein 60 (HSP60)-containing cells in control and cleft-affected supportive tissue. (**A**) A few HSP60-positive osteocytes (arrows) in bone tissue of a control patient, HSP60 IMH, 200×. (**B**) A few HSP60-positive osteocytes (arrows) in cleft-affected bone tissue, HSP60 IMH, 200×. (**C**) Few to moderate number of HSP60-positive chondrocytes and chondroblasts (arrow) in control patient hyaline cartilage, HSP60 IMH, 200×. (**D**) Moderate to numerous HSP60-positive chondrocytes and chondroblasts in cleft-affected cartilage, HSP60 IMH, 200×.

In general, within bone tissue controls, there were a few (+) HSP60-positive bone cells as the median number for this group (Figure 4A). Three patients had a few (+) HSP60-containing cells, while two patients had few to moderate (+/++) factor-positive cells.

In cleft-affected bone tissue, there was a moderate (++) number of HSP60-positive cells as the median value. One patient had a few (+), one patient had a few to moderate (+/++) and three patients had a moderate (++) number of HSP60-positive bone cells (Figure 4B).

In the cartilage tissue control group, there were a few to moderate (+/++) number of HSP60-positive chondrocytes as the median number (Figure 4C). Three patients had few to moderate (+/++) HSP60-containing cells, one patient had a moderate (++) number and another patient had moderate to numerous (++/+++) HSP60-positive cells.

In cleft-affected cartilage, the median number of HSP60-positive cells was a moderate (++) number. One patient had a few (+) HSP60-positive cells, one patient had few to moderate (+/++), two patients had a moderate (++) number and one patient had moderate to numerous (++/+++) factor-positive cells (Figure 4D).

There were no statistically significant differences in the number of HSP60-positive cells between bone controls and cleft-affected bone tissue (*p* = 0.095) and between cartilage controls and cleft-affected cartilage (*p* = 1.000).

#### 3.2.4. HSP70

**Figure 5:**
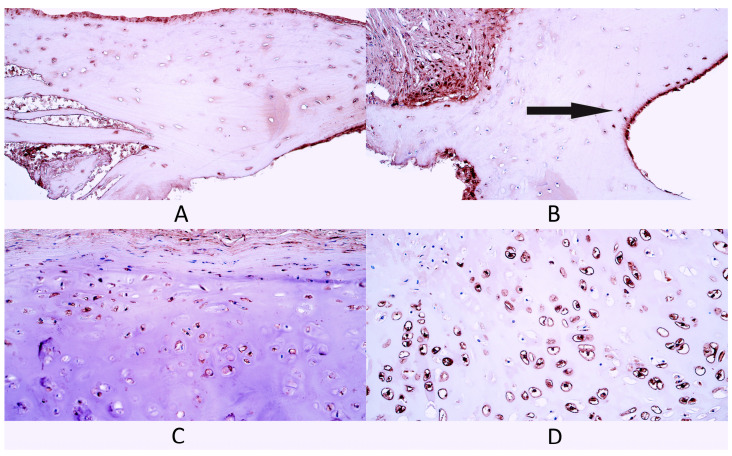
Immunohistochemistry of heat shock protein 70 (HSP70)-containing cells in control and cleft-affected supportive tissue. (**A**) Moderate number of HSP70-positive osteocytes in control bone tissue, HSP70 IMH, 200×. (**B**) A few HSP70-positive bone cells (arrow) in cleft-affected bone tissue, HSP70 IMH, 200×. (**C**) Moderate number of HSP70-positive chondrocytes in control hyaline cartilage, HSP70 IMH, 200×. (**D**) Numerous HSP70-positive chondrocytes in cleft-affected cartilage, HSP70 IMH, 200×.

In bone controls, the median number of HSP70-positive cells was a few (+). One patient had a rare occurrence of HSP70-positive cells, three patients had a few (+) HSP70-positive cells and one patient had a moderate (++) number of HSP70-positive cells (Figure 5A).

In cleft-affected bone tissue, there were few to moderate (+/++) HSP7-positive bone cells, which was the median value for this group. Two patients had a few (+) factor-positive cells, while two patients had few to moderate (+/++) and one patient had a moderate number (++) of HSP70-containing bone cells (Figure 5B).

In cartilage controls, the median number of HSP70-containing cells was few to moderate (+/++). Three patients had a few to moderate (+/++) number of factor-positive cells, while two patients had a moderate (++) number (Figure 5C).

In cleft-affected cartilage, there were numerous (+++) HSP70-containing cartilage cells, which was the median value for this group. Two patients had moderate to numerous (++/+++) factor-positive cells and three patients had numerous (+++) factor-positive cells (Figure 5D).

A statistically significant difference was not found in the number of HSP70-containing cells between bone controls and cleft-affected bone (*p* = 0.310). A statistically significant difference was calculated between cartilage controls and cleft-affected cartilage (*p* = 0.008).

#### 3.2.5. LL-37

**Figure 6:**
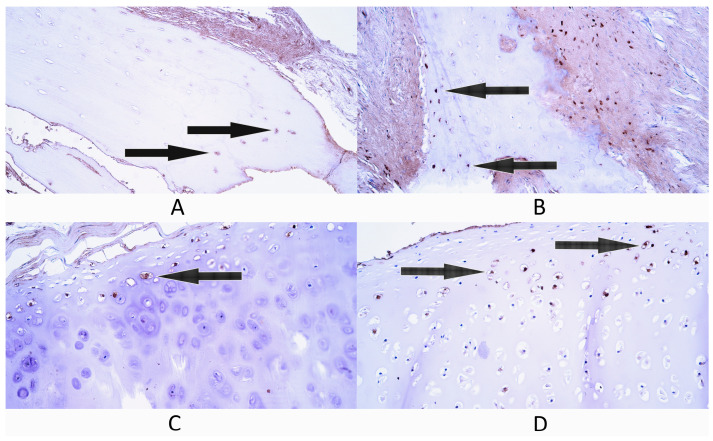
Immunohistochemistry of cathelicidin (LL-37)-containing cells in control and cleft-affected supportive tissue. (**A**) A few LL-37-positive osteocytes (arrows) in control bone tissue, LL-37 IMH, 200×. (**B**) Few to moderate number of LL-37-positive osteocytes (arrows) in cleft-affected bone tissue, LL-37 IMH, 200×. (**C**) A few LL-37-positive chondrocytes (arrow) in control cartilage tissue, LL-37 IMH, 200×. (**D**) Moderate number of LL-37-positive chondrocytes (arrows) in cleft-affected cartilage, LL-37 IMH, 200×.

In bone controls, there was a rare occurrence (0/+) of LL-37-positive cells, which was the median value for this group. Four patients had a rare occurrence of LL-37-positive cells, while one control patient did not have any (0) LL-37-positive bone cells (Figure 6A).

In general, within the cleft-affected bone tissue group, there was a rare occurrence (0/+) of LL-37-positive bone cells. Four patients had a rare occurrence (0/+) of factor-positive cells, while one patient had a few (+) LL-37-containing cells (Figure 6B).

In cartilage controls, the median value of LL-37-positive cells was few to moderate (+/++). Four patients had a few to moderate (+/++) number of LL-37-positive cells, while one patient had a moderate (++) number of LL-37-positive cells (Figure 6C).

In general, within the cleft-affected cartilage group, there were a few (+) LL-37-containing cartilage cells. One patient had no (0) LL-37-positive cells, one patient had a rare occurrence (0/+), two patients had a few (+) and one patient had a moderate (++) number of LL-37-positive cells (Figure 6D).

There were no statistically significant differences found in the number of LL-37-positive cells between the bone tissue control group and cleft-affected bone tissue (*p* = 0.421) and between cartilage controls and cleft-affected cartilage (*p* = 0.095).

#### 3.2.6. Col-I

**Figure 7:**
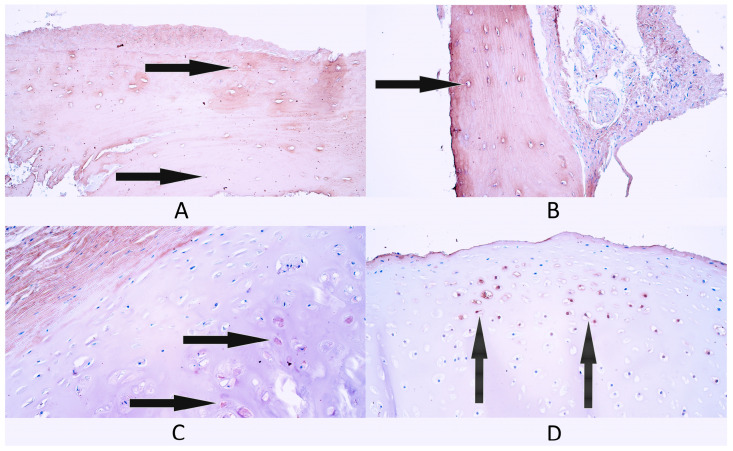
Immunohistochemistry of type I collagen (Col-I)-containing cells in control and cleft-affected supportive tissue. (**A**) A rare occurrence of Col-I-positive osteocytes (arrows) in control bone tissue, Col-I IMH, 200×. (**B**) A few Col-I-positive osteocytes (arrows) in cleft-affected bone tissue, Col-I IMH, 200×. (**C**) A few Col-I-positive chondrocytes (arrows) in control hyaline cartilage, Col-I IMH, 200×. (**D**) Few to moderate number of Col-I-positive chondrocytes (arrows) in cleft-affected hyaline cartilage, Col-I IMH, 200×.

In general, the bone control tissue group had a few (+) Col-I-positive cells, which was the median value in this group. Two patients had a rare occurrence (0/+), two patients had a few (+) and one patient had few to moderate (+/++) Col-I-positive cells (Figure 7A).

The median number of Col-I-positive cells in cleft-affected bone tissue was a moderate (++) number. One patient had a few (+) Col-I-positive bone cells, another patient had a few to moderate (+/++) number and three patients had a moderate (++) number of Col–I-positive cells (Figure 7B).

In general, cartilage controls had a few (+) Col-I-positive cells. Two patients in the cartilage control group had a rare occurrence (0/+) of Col-I-positive cells, two patients had a few (+) and one control patient had a few to moderate (+/++) Col-I-positive cartilage cells (Figure 7C).

In general, there was a rare occurrence (0/+) of Col-I-positive cartilage cells in the cleft-affected cartilage group. A rare occurrence (0/+) of Col-I-positive cartilage cells was seen in three patients, one patient had a few (+) positive cells and one patient had a few to moderate (+/++) positive chondrocytes (Figure 7D).

A statistically significant difference was calculated in the number of Col-I-positive cells between the bone control group and cleft-affected bone tissue (*p* = 0.032). There was no statistically significant difference in the number of Col-I-positive cells between cartilage controls and cleft-affected cartilage (*p* = 0.690).

#### 3.2.7. BMP-2/4

**Figure 8:**
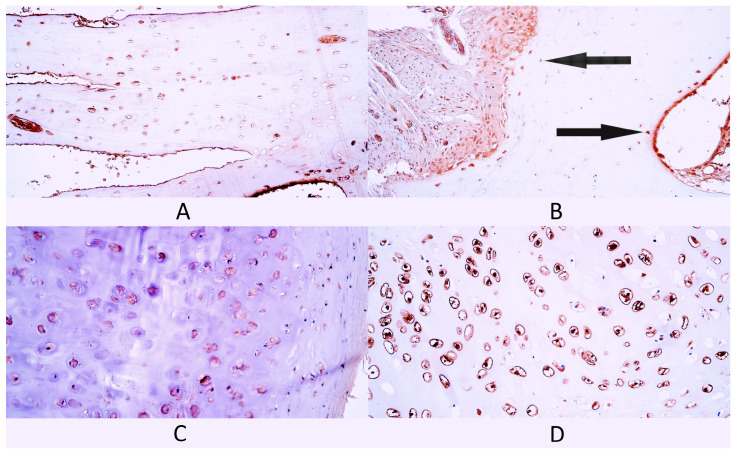
Immunohistochemistry of bone morphogenetic protein 2/4 (BMP-2/4)-containing cells in control and cleft-affected supportive tissue. (**A**) Moderate number of BMP-2/4-positive osteocytes in control bone tissue, BMP-2/4 IMH, 200×. (**B**) A few BMP-2/4-positive osteocytes (arrows) in cleft-affected bone tissue, BMP-2/4 IMH, 200×. (**C**) Moderate number of BMP-2/4-positive chondrocytes in control hyaline cartilage, BMP-2/4 IMH, 200×. (**D**) Numerous to abundant BMP-2/4-positive chondrocytes in cleft-affected hyaline cartilage, BMP-2/4 IMH, 200×.

In the bone control group, there was a moderate (++) number of BMP-2/4-positive bone cells, which was the median value in this group. Two patients had a few (+) BMP-2/4-positive bone cells, while three patients had a moderate number (++) of factor-positive cells (Figure 8A).

In cleft-affected bone tissue, the median number of BMP-2/4-positive cells was a moderate (++) number. Two patients had few to moderate (+/++) factor-positive cells, one patient had a moderate (++) number and two patients had moderate to numerous (++/+++) BMP-2/4-positive cells (Figure 8B).

In the cartilage control group, the median number of BMP-2/4-positive cartilage cells was a moderate (++) number. Two patients had few to moderate (+/++) BMP2/4-positive cells, one patient had a moderate number (++) and two patients had moderate to numerous (++/+++) factor-positive cells Figure 8C).

In the cleft-affected cartilage tissue group, the median number of BMP-2/4-positive cartilage cells was moderate to numerous (++/+++). Each patient had a slightly different number of BMP-2/4-positive cells. One patient had a few to moderate (+/++) number of factor-positive cells, another patient had a moderate (++) number, another had moderate to numerous (++/+++), another had numerous (+++) and one had numerous to abundant (+++/++++) BMP-2/4-positive cells (Figure 8D).

There were no statistically significant differences in the number of BMP-2/4-positive cells between bone tissue controls and cleft-affected bone tissue (*p* = 0.310) and between cartilage controls and cleft-affected cartilage tissue (*p* = 0.310).

The summary of Gal-10-, NF-κB p65-, HSP60-, HSP70-, LL-37-, Col-I- and BMP-2/4-positive cells within each slide can be found in Table 1.

### 3.3. Correlations

In the bone control group, four statistically significant very strong positive correlations were found as follows: between the number of Gal-10-positive cells and BMP-2/4-positive cells (r_s_ = 1.000; *p* < 0.001), between NF-κB p65-positive cells and HSP60-positive cells (r_s_ = 1.000; *p* < 0.001), between Gal-10-positive cells and Col-I-positive cells (r_s_ = 0.913; *p* = 0.030) and between Col-I- and BMP-2/4-containing cells (r_s_ = 0.913; *p* = 0.030).

In cleft-affected bone tissue, one statistically significant correlation between factor-positive cells was found—a statistically significant very strong negative correlation between Gal-10-containing cells and Col-I-containing cells (r_s_ = −1.000; *p* < 0.001).

In the cartilage control group, two statistically significant correlations were calculated. Statistically significant very strong negative correlations were found between the number of HSP70-positive cells and BMP-2/4-positive cells (r_s_ = −0.913; *p* = 0.030) and between the number of HSP60-positive cells and Col-I-positive cells (r_s_ =−0.884; *p* = 0.047).

In cleft-affected cartilage, two statistically significant correlations were calculated. Statistically significant strong positive correlations were found between the number of HSP60-positive cells and LL-37-positive cells (r_s_ = 1.000; *p* < 0.001) and between the number of Gal-10-positive cells and BMP-2/4-positive cells (r_s_ = 0.894; *p* = 0.041).

## 4. Discussion

### 4.1. Immunohistochemistry Evaluation

Statistically significant differences were found between control cartilage and cleft-affected tissue groups in the number of HSP70-positive cartilage cells. The significant increase in HSP70-positive cells within cleft-affected cartilage in comparison to control cartilage tissue could indicate the potential increase in protective action, which might be enhanced due to the presence of the cleft. The presence of orofacial clefts has been associated with increased levels of pro-inflammatory cytokines and increased local inflammatory response due to the disruption of orofacial tissue homeostasis [59,60]. HSP70 provides protective action against inflammation and increases the production of anti-inflammatory cytokines [61,62]. It seems that HSP70 could provide this protective function within cleft-affected cartilage. Interestingly, the relative number of HSP70-positive cells did not differ significantly between control bone and cleft-affected bone tissue, which might mean that there could be differences in HSP70 production between different supportive tissue types and in different orofacial regions. It has been suggested that increased production of HSP70 could be associated with the severity of inflammation in the nasal cavity during chronic rhinosinusitis [63]. Patients with cleft lip and palate have a higher likelihood of developing sinusitis and nasal airway obstruction due to the defect in nasal cartilage [64,65], which again enhances the presence of local inflammation. As the cleft-affected cartilage was taken from the nasal septum of cleft lip and palate patients, this could explain the increased number of HSP70-positive chondrocytes as a compensatory mechanism to moderate inflammation in this craniofacial region. Additional research about the presence of HSP70 in the surrounding mucosal tissue could also be helpful to better explain the immunomodulating and protective action of this heat shock protein in cleft-affected tissue.

Another statistically significant difference in immunoreactivity was found between control bone tissue and cleft-affected bone in the number of Col-I-positive osteocytes. The significant increase in Col-I-positive osteocytes in cleft-affected bone tissue in comparison to controls could indicate increased osteocyte functional activity within cleft-affected bone. Although Col-I in bone tissue is mainly produced by osteoblasts, it has been noted that osteocytes can also produce Col-I [43,66]. Osteocytes can resorb the surrounding bone matrix with Col-I, produce new Col-I and deposit it around themselves within the perilacunar region, which could be an important mechanism for maintaining bone homeostasis [67]. Theoretically, osteocytes could increase collagen production in the cleft-affected bone as a response to increased bone remodeling activity, possibly due to surrounding inflammation and the local tissue defect. In the case of cleft lip and palate, correct bone growth and regeneration are disturbed due to the defect during the jaw development process [68]. Osteocytes could try to compensate for the presence of the cleft by increasing their metabolic activity and collagen I production to maintain bone homeostasis together with other bone cells. Additionally, osteocytes themselves can actively participate in local inflammation by producing different types of cytokines and immunomodulating proteins, which can affect collagen production, bone growth and degeneration [69]. Interestingly, Col-I-positive chondrocytes were also found in cleft-affected cartilage, although the number of Col-I-positive cells did not differ significantly from the controls. Col-I in hyaline cartilage was detected only within chondrocytes and chondroblasts, but not within the cartilage matrix. Expression of Col-I on the transcriptome level has been documented in different hyaline cartilage types and it has been associated with differences in cartilage differentiation and growth, depending on the anatomical location [70]. Our results could indicate that Col-I production on the proteomic level can also be detected in some hyaline cartilage cells, which could imply Col-I’s regulatory role in hyaline cartilage growth, although the exact mechanisms are unclear.

Although no statistically notable differences were found between controls and cleft-affected supportive tissue in the number of Gal-10-, NF-κB p65-, HSP60-, LL-37- and BMP-2/4-positive cells, which could indicate that these proteins might not be significantly involved in cleft-affected supportive tissue homeostasis regulation. The presence of these factors in both relatively healthy and cleft-affected bone and hyaline cartilage most likely indicates their possible necessity and importance in regulating bone and cartilage homeostasis in general.

Gal-10-positive bone and cartilage cells have not yet been described, although other galectins have been notified in human supportive tissue. For example, galectin-1 (Gal-1) induces cartilage degeneration by inducing nuclear factor-kappa beta (NF-κB) mediated inflammation [71] and galectin-3 (Gal-3), which are involved with bone regeneration, vascularization and inflammatory processes [72]. This could mean that Gal-10 could have a similar functional activity in bone and cartilage tissue, although the exact interactions remain uncertain.

NF-κB p65 is actively involved in supportive tissue metabolism and inflammation [73,74], but our research results indicate that the presence of NF-κB p65 is similar in both healthy and cleft-affected supportive tissue, which would mean that most likely other factors are more involved in cleft supportive tissue growth regulation. Although it is difficult to exactly quantify, hyaline cartilage of both controls and cleft patients had relatively more NF-κB p65-positive cells than cleft-affected and control bone tissue, indicating differences in NF-κB p65 functional activity depending on the supportive tissue type.

Similarly, the presence of HSP60-positive bone and cartilage cells might indicate possible activity in regulating supportive tissue metabolism, although there were no statistically significant differences between control groups and cleft-affected tissue groups. HSP60 in bone tissue has been associated with increased osteoclastic bone resorption activity [26], while a decrease in HSP60 in inflamed osteoarthritic cartilage has been noted [20]. The exact role of HSP60 in craniofacial supportive tissue remains unclear and most likely it is not associated with bone metabolism defects within cleft-affected supportive tissue.

LL-37 immunoreactivity was more pronounced in cartilage tissue than bone, although there were no statistically significant differences between the control groups and patient groups for each respective supportive tissue type. LL-37 effects on tissue growth regulation and regeneration have been previously emphasized more in bone tissue [31] than cartilage, although LL-37 might have protective properties in cartilage, as seen in murine models [75]. Our results could indicate that LL-37 could be involved in orofacial supportive tissue metabolism, while the exact role and interactions in cleft-affected supportive tissue are unclear.

BMP-2/4 immunoreactivity was similar in all evaluated control and patient tissue groups, indicating that BMP-2/4 most likely is not significantly involved in tissue growth and formation differences between the controls and cleft-affected tissue in evaluated individuals. BMP signaling disturbances, especially the loss of BMP signaling activity, have been previously associated with cleft lip and palate formation [76]. In our evaluated tissue samples, the similar presence of BMP-2/4 in all groups most likely indicates that other signaling pathways and molecular factors could be more involved in affecting the disturbances seen in cleft-affected tissue metabolism and homeostasis.

### 4.2. Correlation Analysis

Multiple statistically significant correlations were identified between the evaluated factors in the bone tissue control group. The very strong positive correlation between Gal-10-positive osteocytes and BMP-2/4-positive osteocytes could indicate a possible interaction between these factors. Although Gal-10 activity has not been previously connected with BMP signaling, other galectins like Gal-3 [77], Gal-8 [78] and Gal-9 [79] can indirectly interact with BMP signaling and regulate multiple aspects of tissue growth and differentiation, including bone tissue metabolism. This possibly could mean that Gal-10, like other galectins, could have a similar function in healthy bone tissue. Another statistically notable very strong correlation was calculated between NF-κB p65-positive osteocytes and HSP60-positive osteocytes. It has been previously documented that HSP60 can indirectly promote the NF-κB signaling pathway to regulate cell survival in cancer cells and prevent apoptosis [80]. There could be a possibility that a similar pattern could be present in relatively healthy bone tissue where both HSP60 and NF-κB signaling could regulate osteocyte survival. The statistically significant very strong correlation between Gal-10-positive osteocytes and Col-I-positive osteocytes was noted. Although the exact interaction between Gal-10 and Col-I is unclear, previous research has shown that galectins, including Gal-10, can induce the production of matrix metalloproteinases (MMPs), which can destroy different components of extracellular matrix including collagens [81]. This could mean that Gal-10 could affect the bone tissue remodeling process and Col-I production in bone tissue, while the exact mechanism is unclear. Another very strong positive correlation was calculated between Col-I-containing osteocytes and BMP-2/4-positive osteocytes. This corresponds with previously known information about BMP signaling inductive effects on Col-I production [76].

Only one statistically significant very strong negative correlation was calculated in the cleft-affected bone tissue group between Gal-10-positive osteocytes and Col-I-positive osteocytes. Interestingly, this correlation was completely opposite to the correlation between Gal-10- and Col-I-positive cells in bone tissue controls, which might indicate a disruption in the bone remodeling process within cleft-affected bone tissue when compared to relatively healthy bone. The possible mechanism of interaction could relate to galectin-induced MMP production [81], which might affect Col-I formation and production within cleft-affected bone tissue. The fact that there are fewer correlations seen in cleft-affected bone tissue than in control bone tissue could indicate differences in molecular signaling mechanisms, possibly due to the presence of orofacial cleft-affected tissue.

A couple of statistically significant very strong negative correlations between some of the evaluated factors were found in the control hyaline cartilage group. The very strong negative correlation between HSP70-positive chondrocytes and BMP-2/4-positive chondrocytes could mean that HSP70 might affect BMP signaling activity within hyaline cartilage. Previous research has shown that HSP70 could affect the growth differentiation of supportive tissue cells like osteocytes and chondrocytes by stimulating BMP signaling activity through increased BMP-2 expression, but the exact mechanism is unknown [82]. The results in our control hyaline cartilage indicate the opposite. Production of HSP70 in relatively normal hyaline cartilage could be a compensatory protective mechanism to maintain tissue homeostasis as a reaction to surrounding stressors within the internal and external environment that could affect cartilage growth and function. The statistically significant very strong negative correlation between HSP60-positive chondrocytes and Col-I-positive chondrocytes could indicate that HSP60 might negatively affect normal chondrocyte metabolism and extracellular matrix production, although the opposite has been documented in previous research in that HSP60 activity in inflamed hyaline cartilage damaged by osteoarthritis helps to prevent the loss of cartilage matrix [20]. This characteristic could be specific for the control group individuals and the anatomical localization of cartilage used for our study, but additional research about the distribution of HSP60 in hyaline cartilage from different anatomical localizations could help to clarify this information.

Some statistically significant correlations were calculated in the cleft-affected hyaline cartilage group. The statistically significant very strong positive correlation between HSP60-positive chondrocytes and LL-37-positive chondrocytes is an interaction that has not been described before within hyaline cartilage. Interestingly, the connection between HSP60 and LL-37 has been documented in Coxsackievirus B3-induced myocarditis, where LL-37 reduces transmission of the virus and decreases HSP60-induced cell apoptosis of cardiomyocytes [83]. Although it is difficult to fully comprehend the possible protective action of LL-37 and HSP60 in cleft-affected cartilage, it might be a possibility that LL-37 might also affect chondrocyte apoptosis in cleft-affected cartilage by targeting and affecting HSP60 function. Another statistically significant very strong correlation was calculated between Gal-10-positive chondrocytes and BMP-2/4-positive chondrocytes, which might indicate that Gal-10 could affect BMP signaling within cleft-affected hyaline cartilage. Previous studies have not described the possible connection between BMP signaling and Gal-10, although it has been previously notified that other galectins like Gal-3 could possibly interact with different supportive tissue growth factors and signaling pathways, like BMP signaling during bone tissue growth and differentiation [72], but the interaction between Gal-10 and BMP-2/4 remains enigmatic. A similar correlation was seen in bone tissue controls, which could indicate some homeostasis regulation similarities between the two supportive tissue types.

### 4.3. Limitations of the Study

One of the limitations of this study is the implementation of only immunohistochemistry for the detection of Gal-10, NF-κB p65, HSP60, HSP70, LL-37, Col-I and BMP-2/4 protein-containing cells within control and patient groups. The use of additional study methods and techniques could provide additional insights into the functional role of these regulatory factors. Immunohistochemistry with the use of the semiquantitative counting method is used for direct visualization of immunopositive cells, but it cannot evaluate exact concentrations of detected proteins within tissue, where other methods like enzyme-linked immunosorbent assay (ELISA) and radioimmunohistochemistry could be a valuable addition. Other methods for determining transcriptomic or genetic differences within evaluated study groups like in situ hybridization or genetic analysis could also improve the assessment of evaluated factors.

This research work is unique because of the use of human tissue for immunohistochemistry and the availability of such tissue is limited due to ethical considerations and practical difficulties in accessing the necessary amount of tissue from children during surgical manipulations. Although an alternative could be the evaluation of factors within orofacial tissue from animals, the expression and distribution of proteins within animal specimens might not be equivalent to the results seen in human tissue due to species-specific differences. Although there are available datasets from animal models, tissue from experimental animals like mice is typically not directly comparable to human tissue due to anatomical, physiological and genetic differences, which could significantly alter the final comparison. Information gathered from previous studies with animal models can be valuable in describing and comparing the interactions between the evaluated proteins, but this information needs to be taken with care to prevent unadvised and generalized conclusions that might not be accurate for human tissue.

Another limitation is the relatively small number of subjects within the patient and control groups. Although some supportive tissue could be gathered during cleft-correcting surgery, there were multiple limitations in accessing any tissue for research purposes. The ethical aspect of this research limits the availability of orofacial supportive tissue, especially for patients who are not affected by orofacial clefts. The formation of the control group is complicated due to the limited access to relatively healthy supportive tissue needed for comparison with cleft-affected tissue groups. The tissue available from the historical collection of the RSU Institute of Anatomy and Anthropology was the closest available tissue that could be used for the comparison with the cleft-affected cartilage patient group, which was not affected by significant pathological conditions. It is very difficult to obtain any relatively healthy supportive tissue from a significant number of children due to ethical considerations and the presence of very specific surgical indications in obtaining said relatively healthy tissue for control groups. Unfortunately, the relatively small number of patients within each group can affect the statistical evaluation of immunoreactivity and correlations. A larger number of patients would improve the statistical power for comparisons of evaluated factors within each group and prevent the effect of data outliers. Due to the limited availability of human tissue, the minimum requirements for correct statistical assessment have been met to finalize this research work.

A possible future research subject could be the comparison of immunomodulation, protection and regeneration factors in different types of orofacial clefts based on cleft severity like complete or incomplete orofacial cleft, or by obtained tissue type, including soft tissue. For now, there are limitations in obtaining the necessary number of patients, which could be subdivided based on cleft type, severity and other parameters. Forming a significantly large study group of cleft patients and controls is time-consuming and may take multiple years until morphological research studies are fully accomplished.

## 5. Conclusions

1. The statistically significant increase in HSP70-positive chondrocytes in cleft-affected cartilage could indicate that HSP70 could provide protective action in cleft-affected cartilage against stressors like inflammation possibly by enhancing the production of anti-inflammatory factors within the cartilage due to the presence of orofacial cleft and disrupted tissue homeostasis.
2. The statistically significant increase in Col-I-positive osteocytes in cleft-affected bone tissue could indicate increased osteocyte functional activity, which might be explained by increased bone remodeling capacity due to the presence of a tissue defect caused by the orofacial cleft.
3. Within evaluated bone tissue groups, multiple statistically significant positive correlations were detected in the bone tissue control group, mainly involving Gal-10, BMP-2/4 and Col-I, while cleft-affected bone tissue had a single statistically significant negative correlation between the number of Gal-10- and Col-I-positive osteocytes, indicating notable differences in factor activity and interactions between the evaluated bone tissue groups. Similarly, differences in statistically significant correlations between factor-positive cells were found in cleft tissue groups—within hyaline cartilage controls, negative correlations were detected involving heat shock proteins, while cleft-affected cartilage had positive correlations, which could mean that molecular interactions between the evaluated factors in orofacial supportive tissue are changed due to the presence of orofacial cleft.

## Figures and Tables

**Table 1:** Immunoreactivity of Gal-10, NF-κB p65, HSP60, HSP70, LL-37, Col-I and BMP-2/4 in supportive tissue controls and cleft-affected cartilage.

	Patient	Gal-10	NF-κB p65	HSP60	HSP70	LL-37	Col-I	BMP-2/4
Bone controls	1	+	+	+/++	++	0/+	+	++
	2	+	0/+	+	+	0/+	+/++	++
	3	0/+	0/+	+	0/+	0/+	0/+	+
	4	+	+	+/++	+	0	+	++
	5	0/+	0/+	+	+	0/+	0/+	+
	Median	+	0/+	+	+	0/+	+	++
Cleft- affected bone tissue	1	+	+	+	+	0/+	++	++/+++
	2	+/++	+	++	+/++	+	+/++	++
	3	+	+	+/++	+/++	0/+	++	+/++
	4	+	+/++	++	+	0/+	++	+/++
	5	++	+/++	++	++	0/+	+	++/+++
	Median	+	+	++	+/++	0/+	++	++
Cartilage controls	1	++/+++	++/+++	++/+++	++	++	0/+	+/++
	2	+/++	++	+/++	+/++	+/++	+	++/+++
	3	++	+/++	+/++	+/++	+/++	+/++	++/+++
	4	++	++	+/++	++	+/++	+	+/++
	5	++	+/++	++	+/++	+/++	0/+	++
	Median	++	++	+/++	+/++	+/++	+	++
Cleft- affected hyaline cartilage	1	+/++	+	+/++	+++	0/+	+	+/++
	2	+/++	+++	+	+++	0	0/+	++/+++
	3	+++	+++	++/+++	+++	++	+/++	+++/++++
	4	++	+++	++	++/+++	+	0/+	+++
	5	+/++	+++	++	++/+++	+	0/+	++
	Median	+/++	+++	++	+++	+	0/+	++/+++

Abbreviations: Gal-10—galectin-10, NF-κB p65—nuclear factor kappa-light-chain-enhancer of activated B cells protein 65, HSP60—heat shock protein 60, HSP70—heat shock protein 70, LL-37—cathelicidin, Col-I—type I collagen, BMP-2/4—bone morphogenetic protein 2/4, 0—no factor-positive cells, 0/+—a rare occurrence of factor-positive cells, +—a few factor-positive cells, +/++—few to moderate number of factor-positive cells, ++—a moderate number of factor-positive cells, ++/+++—moderate to numerous factor-positive cells, +++—numerous factor-positive cells, +++/++++—numerous to abundant number of factor-positive cells.

## Data Availability

Due to ethical concerns, data are not publicly available. Data are available at the request of the corresponding author.

## References

[1] Vyas T., Gupta P., Kumar S., Gupta R., Gupta T., Singh H. (2020). Cleft of Lip and Palate: A Review. J. Fam. Med. Prim. Care.

[2] Smarius B., Loozen C., Manten W., Bekker M., Pistorius L., Breugem C. (2017). Accurate Diagnosis of Prenatal Cleft Lip/Palate by Understanding the Embryology. World J. Methodol..

[3] Farber S.J., Maliha S.G., Gonchar M.N., Kantar R.S., Shetye P.R., Flores R.L. (2019). Effect on Facial Growth of the Management of Cleft Lip and Palate. Ann. Plast. Surg..

[4] Shi B., Losee J.E. (2015). The Impact of Cleft Lip and Palate Repair on Maxillofacial Growth. Int. J. Oral Sci..

[5] Su J. (2018). A Brief History of Charcot-Leyden Crystal Protein/Galectin-10 Research. Molecules.

[6] Melo R.C.N., Wang H., Silva T.P., Imoto Y., Fujieda S., Fukuchi M., Miyabe Y., Hirokawa M., Ueki S., Weller P.F. (2020). Galectin-10, the Protein That Forms Charcot-Leyden Crystals, Is Not Stored in Granules but Resides in the Peripheral Cytoplasm of Human Eosinophils. J. Leukoc. Biol..

[7] Pichler K.M., Weinmann D., Schmidt S., Kubista B., Lass R., Martelanz L., Alphonsus J., Windhager R., Gabius H.-J., Toegel S. (2021). The Dysregulated Galectin Network Activates NF-ΚB to Induce Disease Markers and Matrix Degeneration in 3D Pellet Cultures of Osteoarthritic Chondrocytes. Calcif. Tissue Int..

[8] Marsich E., Mozetic P., Ortolani F., Contin M., Marchini M., Vetere A., Pacor S., Semeraro S., Vittur F., Paoletti S. (2008). Galectin-1 in Cartilage: Expression, Influence on Chondrocyte Growth and Interaction with ECM Components. Matrix Biol..

[9] Giridharan S., Srinivasan M. (2018). Mechanisms of NF-ΚB P65 and Strategies for Therapeutic Manipulation. J. Inflamm. Res..

[10] Parrondo R., de las Pozas A., Reiner T., Rai P., Perez-Stable C. (2010). NF-ΚB Activation Enhances Cell Death by Antimitotic Drugs in Human Prostate Cancer Cells. Mol. Cancer.

[11] Boyce B.F., Yao Z., Xing L. (2010). Functions of Nuclear Factor ΚB in Bone. Ann. N. Y. Acad. Sci..

[12] Li Y., Li A., Strait K., Zhang H., Nanes M.S., Weitzmann M.N. (2007). Endogenous TNFα Lowers Maximum Peak Bone Mass and Inhibits Osteoblastic Smad Activation through NF-ΚB. J. Bone Miner. Res..

[13] Kobayashi H., Hirata M., Saito T., Ito S., Hosaka Y., Chung U.-I., Kawaguchi H. (2013). NF-ΚB Family Member RELA/P65 in Chondrocytes Controls Skeletal Growth and Osteoarthritis Development by Inhibiting Chondrocyte Apoptosis. Osteoarthr. Cartil..

[14] Caruso Bavisotto C., Alberti G., Vitale A.M., Paladino L., Campanella C., Rappa F., Gorska M., Conway de Macario E., Cappello F., Macario A.J.L. (2020). Hsp60 Post-Translational Modifications: Functional and Pathological Consequences. Front. Mol. Biosci..

[15] Okamoto T., Yamamoto H., Kudo I., Matsumoto K., Odaka M., Grave E., Itoh H. (2017). HSP60 Possesses a GTPase Activity and Mediates Protein Folding with HSP10. Sci. Rep..

[16] Domínguez-Horta M.d.C., Serrano-Díaz A., Hernández-Cedeño M., Martínez-Donato G., Guillén-Nieto G. (2023). A Peptide Derived from HSP60 Reduces Proinflammatory Cytokines and Soluble Mediators: A Therapeutic Approach to Inflammation. Front. Immunol..

[17] Spierings J., van Eden W. (2017). Heat Shock Proteins and Their Immunomodulatory Role in Inflammatory Arthritis. Rheumatology.

[18] Wang F.-S., Wu R.-W., Ko J.-Y., Tai M.-H., Ke H.-C., Yeh D.-W., Wu S.-L., Chen M.-W. (2011). Heat Shock Protein 60 Protects Skeletal Tissue against Glucocorticoid-Induced Bone Mass Loss by Regulating Osteoblast Survival. Bone.

[19] Lian W.-S., Ko J.-Y., Chen Y.-S., Ke H.-C., Wu S.-L., Kuo C.-W., Wang F.-S. (2018). Chaperonin 60 Sustains Osteoblast Autophagy and Counteracts Glucocorticoid Aggravation of Osteoporosis by Chaperoning RPTOR. Cell Death Dis..

[20] Ko J.-Y., Sun Y.-C., Li W.-C., Wang F.-S. (2016). Chaperonin 60 Regulation of SOX9 Ubiquitination Mitigates the Development of Knee Osteoarthritis. J. Mol. Med..

[21] Kim J.Y., Barua S., Huang M.Y., Park J., Yenari M.A., Lee J.E. (2020). Heat Shock Protein 70 (HSP70) Induction: Chaperonotherapy for Neuroprotection after Brain Injury. Cells.

[22] Rosenzweig R., Nillegoda N.B., Mayer M.P., Bukau B. (2019). The Hsp70 Chaperone Network. Nat. Rev. Mol. Cell Biol..

[23] Alharbi B.M., Albinhassan T.H., Alzahrani R.A., Bouchama A., Mohammad S., Alomari A.A., Bin-Jumah M.N., AlSuhaibani E.S., Malik S.S. (2023). Profiling the Hsp70 Chaperone Network in Heat-Induced Proteotoxic Stress Models of Human Neurons. Biology.

[24] Cappucci U., Noro F., Casale A.M., Fanti L., Berloco M., Alagia A.A., Grassi L., Le Pera L., Piacentini L., Pimpinelli S. (2019). The Hsp70 Chaperone Is a Major Player in Stress-Induced Transposable Element Activation. Proc. Natl. Acad. Sci. USA.

[25] Notsu K., Nakagawa M., Nakamura M. (2016). Ubiquitin-like Protein MNSFβ Noncovalently Binds to Molecular Chaperone HSPA8 and Regulates Osteoclastogenesis. Mol. Cell. Biochem..

[26] Hang K., Ye C., Chen E., Zhang W., Xue D., Pan Z. (2018). Role of the Heat Shock Protein Family in Bone Metabolism. Cell Stress Chaperones.

[27] Guo Y., Stampoultzis T., Karami P., Nasrollahzadeh N., Rana V.K., Pioletti D.P. (2024). HSP70—A Key Regulator in Chondrocyte Homeostasis under Naturally Coupled Hydrostatic Pressure-Thermal Stimuli. Osteoarthr. Cartil..

[28] Child D.F., Hudson P.R., Hunter-Lavin C., Mukhergee S., China S., Williams C.P., Williams J.H.H. (2006). Birth Defects and Anti–Heat Shock Protein 70 Antibodies in Early Pregnancy. Cell Stress Chaperones.

[29] Kahlenberg J.M., Kaplan M.J. (2013). Little Peptide, Big Effects: The Role of LL-37 in Inflammation and Autoimmune Disease. J. Immunol..

[30] Yang B., Good D., Mosaiab T., Liu W., Ni G., Kaur J., Liu X., Jessop C., Yang L., Fadhil R. (2020). Significance of LL-37 on Immunomodulation and Disease Outcome. Biomed. Res. Int..

[31] Chinipardaz Z., Zhong J.M., Yang S. (2022). Regulation of LL-37 in Bone and Periodontium Regeneration. Life.

[32] Li L., Peng Y., Yuan Q., Sun J., Zhuang A., Bi X. (2021). Cathelicidin LL37 Promotes Osteogenic Differentiation in Vitro and Bone Regeneration in Vivo. Front. Bioeng. Biotechnol..

[33] Kittaka M., Shiba H., Kajiya M., Fujita T., Iwata T., Rathvisal K., Ouhara K., Takeda K., Fujita T., Komatsuzawa H. (2013). The Antimicrobial Peptide LL37 Promotes Bone Regeneration in a Rat Calvarial Bone Defect. Peptides.

[34] Kapat K., Kumbhakarn S., Sable R., Gondane P., Takle S., Maity P. (2024). Peptide-Based Biomaterials for Bone and Cartilage Regeneration. Biomedicines.

[35] Najmi Z., Kumar A., Scalia A.C., Cochis A., Obradovic B., Grassi F.A., Leigheb M., Lamghari M., Loinaz I., Gracia R. (2020). Evaluation of Nisin and LL-37 Antimicrobial Peptides as Tool to Preserve Articular Cartilage Healing in a Septic Environment. Front. Bioeng. Biotechnol..

[36] Naomi R., Ridzuan P.M., Bahari H. (2021). Current Insights into Collagen Type I. Polymers.

[37] Amirrah I.N., Lokanathan Y., Zulkiflee I., Wee M.F.M.R., Motta A., Fauzi M.B. (2022). A Comprehensive Review on Collagen Type I Development of Biomaterials for Tissue Engineering: From Biosynthesis to Bioscaffold. Biomedicines.

[38] Alcaide-Ruggiero L., Molina-Hernández V., Granados M.M., Domínguez J.M. (2021). Main and Minor Types of Collagens in the Articular Cartilage: The Role of Collagens in Repair Tissue Evaluation in Chondral Defects. Int. J. Mol. Sci..

[39] Brown W.E., Lavernia L., Bielajew B.J., Hu J.C., Athanasiou K.A. (2023). Human Nasal Cartilage: Functional Properties and Structure-Function Relationships for the Development of Tissue Engineering Design Criteria. Acta Biomater..

[40] Sasano Y., Takahashi I., Zhu J.-X., Ohtani H., Mizoguchi I., Kagayama M. (2001). Gene and Protein Expressions of Type I Collagen Are Regulated Tissue-Specifically in Rat Hyaline Cartilages in Vivo. Eur. J. Morphol..

[41] Li Y., Liu Y., Li R., Bai H., Zhu Z., Zhu L., Zhu C., Che Z., Liu H., Wang J. (2021). Collagen-Based Biomaterials for Bone Tissue Engineering. Mater. Des..

[42] Boraschi-Diaz I., Wang J., Mort J.S., Komarova S.V. (2017). Collagen Type I as a Ligand for Receptor-Mediated Signaling. Front. Phys..

[43] Shiflett L.A., Tiede-Lewis L.M., Xie Y., Lu Y., Ray E.C., Dallas S.L. (2019). Collagen Dynamics during the Process of Osteocyte Embedding and Mineralization. Front. Cell Dev. Biol..

[44] Choi J.U.A., Kijas A.W., Lauko J., Rowan A.E. (2022). The Mechanosensory Role of Osteocytes and Implications for Bone Health and Disease States. Front. Cell Dev. Biol..

[45] Sasano Y., Furusawa M., Ohtani H., Mizoguchi I., Takahashi I., Kagayama M. (1996). Chondrocytes Synthesize Type I Collagen and Accumulate the Protein in the Matrix during Development of Rat Tibial Articular Cartilage. Anat. Embryol..

[46] Gagliano N., Carinci F., Moscheni C., Torri C., Pezzetti F., Scapoli L., Martinelli M., Gioia M., Stabellini G. (2010). New Insights in Collagen Turnover in Orofacial Cleft Patients. Cleft Palate Craniofac. J..

[47] Halloran D., Durbano H.W., Nohe A. (2020). Bone Morphogenetic Protein-2 in Development and Bone Homeostasis. J. Dev. Biol..

[48] Alarmo E.-L., Huhtala H., Korhonen T., Pylkkänen L., Holli K., Kuukasjärvi T., Parkkila S., Kallioniemi A. (2013). Bone Morphogenetic Protein 4 Expression in Multiple Normal and Tumor Tissues Reveals Its Importance beyond Development. Mod. Pathol..

[49] Cheng H., Gao X., Huard M., Lu A., Ruzbarsky J.J., Amra S., Wang B., Huard J. (2022). Bone Morphogenetic Protein 4 Rescues the Bone Regenerative Potential of Old Muscle-Derived Stem Cells via Regulation of Cell Cycle Inhibitors. Stem Cell Res. Ther..

[50] Khan M.P., Khan K., Yadav P.S., Singh A.K., Nag A., Prasahar P., Mittal M., China S.P., Tewari M.C., Nagar G.K. (2016). BMP Signaling Is Required for Adult Skeletal Homeostasis and Mediates Bone Anabolic Action of Parathyroid Hormone. Bone.

[51] Yamaguchi H., Swaminathan S., Mishina Y., Komatsu Y. (2023). Enhanced BMP Signaling Leads to Enlarged Nasal Cartilage Formation in Mice. Biochem. Biophys. Res. Commun..

[52] Schmitt B., Ringe J., Häupl T., Notter M., Manz R., Burmester G.-R., Sittinger M., Kaps C. (2003). BMP2 Initiates Chondrogenic Lineage Development of Adult Human Mesenchymal Stem Cells in High-Density Culture. Differentiation.

[53] Miljkovic N.D., Cooper G.M., Marra K.G. (2008). Chondrogenesis, Bone Morphogenetic Protein-4 and Mesenchymal Stem Cells. Osteoarthr. Cartil..

[54] Buile D., Pilmane M., Akota I. (2020). Appearance and Distribution of Tissue Remodellation Factors in the Hard Tissue of Patients Affected by Cleft Lip Palate. Proc. Latv. Acad. Sci. Sect. B Nat. Exact Appl. Sci..

[55] Kauffmann P., Kolle J., Quast A., Wolfer S., Schminke B., Meyer-Marcotty P., Schliephake H. (2024). Two-Stage Palatal Repair in Non-Syndromic CLP Patients Using Anterior to Posterior Closure Is Associated with Minimal Need for Secondary Palatal Surgery. Head Face Med..

[56] Kommineni N., Reddy C.V., Chandra N., Reddy D.S., Kumar A., Reddy M.V. (2014). Mixed Dentition Analysis—Applicability of Two Non-Radiographic Methods for Chennai School Children. J. Int. Soc. Prev. Community Dent..

[57] Hsu S.-M., Raine L., Fanger H. (1981). The Use of Antiavidin Antibody and Avidin-Biotin-Peroxidase Complex in Immunoperoxidase Technics. Am. J. Clin. Pathol..

[58] Pilmane M., Shine J., Iismaa T.P. (1998). Distribution of Galanin Immunoreactivity in the Bronchi of Humans with Tuberculosis. Ann. N. Y. Acad. Sci..

[59] Seidel C.L., Percivalle E., Tschaftari M., Weider M., Strobel K., Willershausen I., Unertl C., Schmetzer H.M., Weber M., Schneider M. (2022). Orofacial Clefts Lead to Increased Pro-Inflammatory Cytokine Levels on Neonatal Oral Mucosa. Front. Immunol..

[60] Pilmane M., Sidhoma E., Akota I., Kazoka D. (2019). Characterization of Cytokines and Proliferation Marker Ki67 in Cleft Affected Lip Tissue. Medicina.

[61] Khandia R., Munjal A.K., Iqbal H.M.N., Dhama K. (2017). Heat Shock Proteins: Therapeutic Perspectives in Inflammatory Disorders. Recent Pat. Inflamm. Allergy Drug Discov..

[62] Jacquier-Sarlin M.R., Fuller K., Dinh-Xuan A.T., Richard M.-J., Polla B.S. (1994). Protective Effects of Hsp70 in Inflammation. Experientia.

[63] Min H.J., Yoon J.-H., Kim C.-H. (2016). HSP70 Is Associated with the Severity of Inflammation in Chronic Rhinosinusitis. Am. J. Rhinol. Allergy.

[64] Sobol D.L., Allori A.C., Carlson A.R., Pien I.J., Watkins S.E., Aylsworth A.S., Meyer R.E., Pimenta L.A., Strauss R.P., Ramsey B.L. (2016). Nasal Airway Dysfunction in Children with Cleft Lip and Cleft Palate: Results of a Cross-Sectional Population-Based Study, with Anatomical and Surgical Considerations. Plast. Reconstr. Surg..

[65] Suzuki H., Yamaguchi T., Furukawa M. (2000). Maxillary Sinus Development and Sinusitis in Patients with Cleft Lip and Palate. Auris Nasus Larynx.

[66] Schlesinger P.H., Blair H.C., Beer Stolz D., Riazanski V., Ray E.C., Tourkova I.L., Nelson D.J. (2020). Cellular and Extracellular Matrix of Bone, with Principles of Synthesis and Dependency of Mineral Deposition on Cell Membrane Transport. Am. J. Physiol. Cell Physiol..

[67] Creecy A., Damrath J.G., Wallace J.M. (2021). Control of Bone Matrix Properties by Osteocytes. Front. Endocrinol..

[68] Martín-del-Campo M., Rosales-Ibañez R., Rojo L. (2019). Biomaterials for Cleft Lip and Palate Regeneration. Int. J. Mol. Sci..

[69] Metzger C.E., Narayanan S.A. (2019). The Role of Osteocytes in Inflammatory Bone Loss. Front. Endocrinol..

[70] Fukunaga T., Yamashiro T., Oya S., Takeshita N., Takigawa M., Takano-Yamamoto T. (2003). Connective Tissue Growth Factor MRNA Expression Pattern in Cartilages Is Associated with Their Type I Collagen Expression. Bone.

[71] Osório J. (2016). Galectin-1 Damages Cartilage via Inflammation. Nat. Rev. Rheumatol..

[72] Iacobini C., Fantauzzi C.B., Pugliese G., Menini S. (2017). Role of Galectin-3 in Bone Cell Differentiation, Bone Pathophysiology and Vascular Osteogenesis. Int. J. Mol. Sci..

[73] Wu S., Flint J.K., Rezvani G., De Luca F. (2007). Nuclear Factor-ΚB P65 Facilitates Longitudinal Bone Growth by Inducing Growth Plate Chondrocyte Proliferation and Differentiation and by Preventing Apoptosis. J. Biol. Chem..

[74] Tak P.P., Firestein G.S. (2001). NF-ΚB: A Key Role in Inflammatory Diseases. J. Clin. Invest..

[75] Chow L.N.Y., Choi K.-Y. (2014). (grace); Piyadasa, H.; Bossert, M.; Uzonna, J.; Klonisch, T.; Mookherjee, N. Human Cathelicidin LL-37-Derived Peptide IG-19 Confers Protection in a Murine Model of Collagen-Induced Arthritis. Mol. Immunol..

[76] Wang R.N., Green J., Wang Z., Deng Y., Qiao M., Peabody M., Zhang Q., Ye J., Yan Z., Denduluri S. (2014). Bone Morphogenetic Protein (BMP) Signaling in Development and Human Diseases. Genes Dis..

[77] Soares L.C., Al-Dalahmah O., Hillis J., Young C.C., Asbed I., Sakaguchi M., O’Neill E., Szele F.G. (2021). Novel Galectin-3 Roles in Neurogenesis, Inflammation and Neurological Diseases. Cells.

[78] Vinik Y., Shatz-Azoulay H., Hiram-Bab S., Kandel L., Gabet Y., Rivkin G., Zick Y. (2018). Ablation of the Mammalian Lectin Galectin-8 Induces Bone Defects in Mice. FASEB J..

[79] Tanikawa R., Tanikawa T., Hirashima M., Yamauchi A., Tanaka Y. (2010). Galectin-9 Induces Osteoblast Differentiation through the CD44/Smad Signaling Pathway. Biochem. Biophys. Res. Commun..

[80] Chun J.N., Choi B., Lee K.W., Lee D.J., Kang D.H., Lee J.Y., Song I.S., Kim H.I., Lee S.-H., Kim H.S. (2010). Cytosolic Hsp60 Is Involved in the NF-ΚB-Dependent Survival of Cancer Cells via IKK Regulation. PLoS ONE.

[81] Sato T., Chiba T., Nakahara T., Watanabe K., Sakai S., Noguchi N., Noto M., Ueki S., Kono M. (2023). Eosinophil-Derived Galectin-10 Upregulates Matrix Metalloproteinase Expression in Bullous Pemphigoid Blisters. J. Dermatol. Sci..

[82] Li C., Sunderic K., Nicoll S.B., Wang S. (2018). Downregulation of Heat Shock Protein 70 Impairs Osteogenic and Chondrogenic Differentiation in Human Mesenchymal Stem Cells. Sci. Rep..

[83] Yang Y., Huang C., Hui L., Song Y., Fu Y., Li M., Yang H., Wu J., Sun J., Xu W. (2023). Cathelicidins Target HSP60 to Restrict CVB3 Transmission via Disrupting the Exosome and Reducing Cardiomyocyte Apoptosis. J. Virol..

